# Immunophenotyping in post-giardiasis functional gastrointestinal disease and chronic fatigue syndrome

**DOI:** 10.1186/1471-2334-12-258

**Published:** 2012-10-14

**Authors:** Kurt Hanevik, Einar K Kristoffersen, Steinar Sørnes, Kristine Mørch, Halvor Næss, Ann C Rivenes, Jørn E Bødtker, Trygve Hausken, Nina Langeland

**Affiliations:** 1 Institute of Medicine, University of Bergen, Bergen, N-5021, Norway; 2 Centre for Tropical Infectious Diseases, Haukeland University Hospital, Bergen, N-5021, Norway; 3 Department of Immunology and Transfusion Medicine, Haukeland University Hospital, Bergen, Norway; 4 The Gade Institute, University of Bergen, Bergen, Norway; 5 Department of Neurology, Haukeland University Hospital, Bergen, Norway; 6 Division of Psychiatry, Haukeland University Hospital, Bergen, Norway; 7 Division of Psychiatry, Haukeland University Hospital, Bergen, Norway

**Keywords:** *Giardia lamblia*, Functional gastrointestinal disorder, Chronic fatigue syndrome, Irritable bowel syndrome, NK-cells, CD8 T-cells

## Abstract

**Background:**

A *Giardia* outbreak was associated with development of post-infectious functional gastrointestinal disorders (PI-FGID) and chronic fatigue syndrome (PI-CFS). Markers of immune dysfunction have given conflicting results in CFS and FGID patient populations. The aim of this study was to evaluate a wide selection of markers of immune dysfunction in these two co-occurring post-infectious syndromes.

**Methods:**

48 patients, reporting chronic fatigue in a questionnaire study, were clinically evaluated five years after the outbreak and grouped according to Fukuda criteria for CFS (n=19) and idiopathic chronic fatigue (n=5) and Rome II criteria for FGIDs (n=54). 22 *Giardia* exposed non-fatigued individuals and 10 healthy unexposed individuals were recruited as controls. Peripheral blood lymphocyte subsets were analyzed by flow cytometry.

**Results:**

In peripheral blood we found significantly higher CD8 T-cell levels in PI-FGID, and significantly lower NK-cell levels in PI-CFS patients. Severity of abdominal and fatigue symptoms correlated negatively with NK-cell levels. A tendency towards lower T-cell CD26 expression in FGID was seen.

**Conclusion:**

Patients with PI-CFS and/or PI-FGID 5 years after *Giardia lamblia* infection showed alterations in NK-cell and CD8-cell populations suggesting a possible immunological abnormality in these conditions. We found no significant changes in other markers examined in this well-defined group of PI-CFS and PI-FGID elicited by a gastrointestinal infection. Controlling for co-morbid conditions is important in evaluation of CFS-biomarkers.

## Background

Infection with the intestinal protozoan parasite *Giardia lamblia* is common in developing countries and is often seen in travelers returning from endemic areas [1]. It is also a frequent cause of waterborne outbreaks in industrialized countries, but it is generally regarded as an uncomplicated infection for which there is effective antibiotic treatment. Although long term abdominal symptoms following acute giardiasis have been observed by clinicians in individual patients for decades, studies on post-giardiasis functional gastrointestinal disorders (FGID) [2] and chronic fatigue [3] have only recently been reported after a waterborne outbreak in Bergen in 2004 [4].

FGID are a group of disorders characterized by recurring or chronic gastrointestinal symptoms without an identifiable disease process [5]. Irritable bowel syndrome (IBS) and functional dyspepsia (FD) are the best described FGID. Fatigue is a frequent symptom in FGID patients [3,6]. One study has found that 14% of IBS patients also have chronic fatigue syndrome (CFS), while six studies report that 35-92% of CFS patients also have IBS [7]. Researchers of FGID as well as CFS [8,9] rarely control for this co-morbidity, even though they are focusing on the same pathophysiologic mechanisms, such as low grade inflammation, immunological dysfunction, neuroendocrine dysfunction, sensory hypersensitivity, sustained stress responses and adverse life events underlying the symptomatology. FGID and CFS share the characteristics of female preponderance, both are diagnoses relying on symptom criteria alone, and in many cases the onset is preceded by an acute infection [10].

Many studies have been reported regarding differences in activation and function in peripheral blood lymphocyte subsets in CFS. These have given inconsistent results, and are reviewed by Natelson et al., with some studies finding altered natural killer (NK)-cell levels and some finding lowered CD4:CD8 ratios, but most studies find normal T, B and NK cell levels in CFS [11]. More recent studies have reported a decrease in the CD56brightCD16- NK-cell subset and increased CD4CD25FoxP3 regulatory T-cells [12], and CD26 expressed on T-cells and NK-cells (marked with CD2) has been put forward as a promising biomarker in CFS [13].

Studies looking at peripheral blood lymphocyte subsets in FGID have not identified differences in regulatory T-cells [14] or lymphocyte subsets, but have found increased levels of B-cells expressing IgG or co-stimulatory molecules CD80 or CD86 and T-cells expressing β7+HLADR+ and CD69+ in IBS-patients compared to controls [15-17].

When the onset of FGID or CFS is associated with an acute infection, it is often termed post-infectious CFS (PI-CFS) [18,19] or FGID (PI-FGID) [20] or in the case of IBS, post-infectious IBS (PI-IBS) [9]. A meta-analysis of PI-IBS estimates that the risk of having IBS one year after an acute gastroenteritis is approximately sixfold [21]. Until recently few studies of FGID and CFS separated between infection-related onset and a less defined onset in these disorders.

The immune responses to *Giardia* infection are known to include both innate and adaptive components [22]. Important roles have been shown for mast cells and IL-6 [23], as well as for B-cell antibody production [24,25]. In mice αβ-TCR-expressing T-cells are required to control infection [26] and CD4 T-cell depletion results in chronic infection [27]. CD8 T-cells seems not to be important for the control of infection in mice, but contribute to the giardiasis related intestinal mucosal injury [28]. Beige mice, which are deficient in NK-cells, have been shown to clear *Giardia* infection equally fast as immunocompetent mice [29].

The present study, performed in a well-defined group of patients with clinically observed post-infectious FGID and CFS after a common eliciting *Giardia* infection, was done to evaluate a wide array of lymphocyte subsets, including many of the previously reported markers of immune dysfunction in CFS and FGID.

## Methods

### Study population

**Figure 1:**
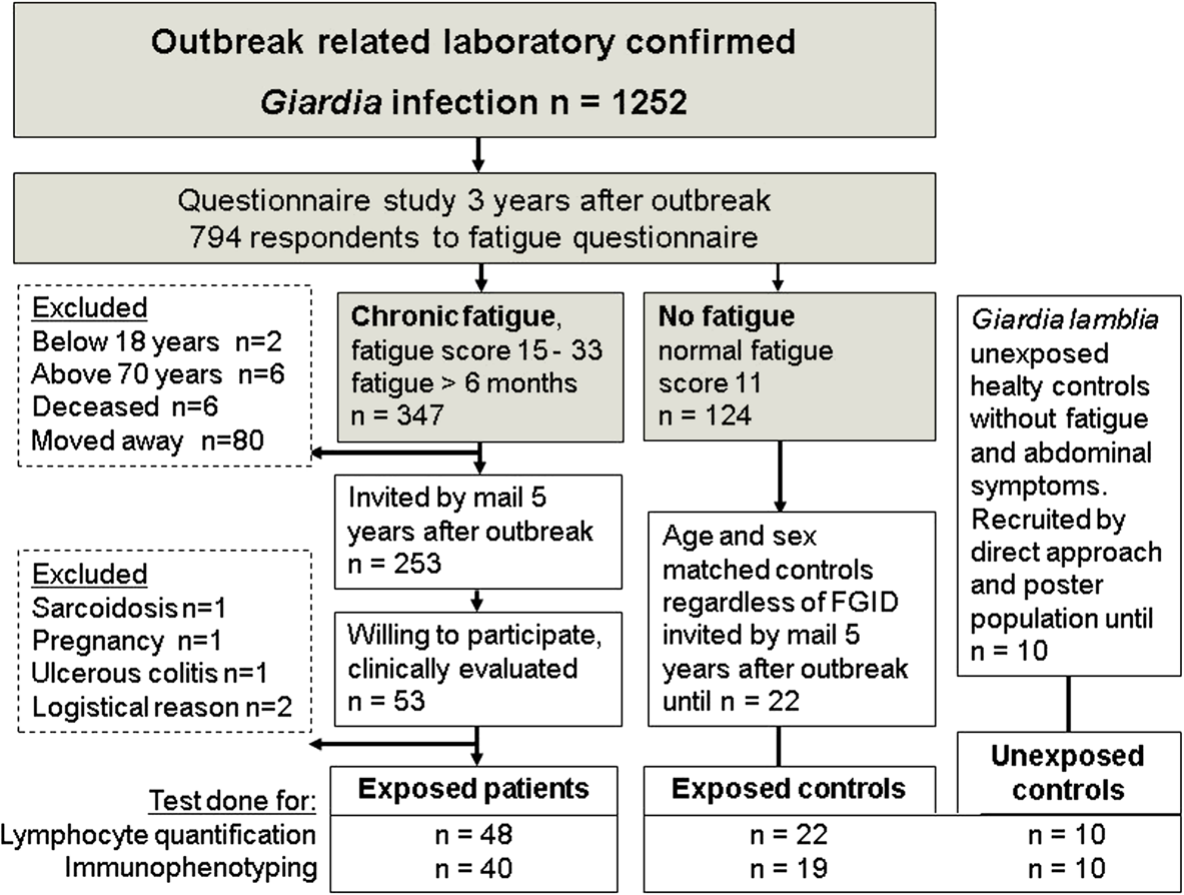
**Study recruitment base and participants.** Participants were recruited based on a mailed questionnaire study regarding fatigue [30] and abdominal complaints [31] to all individuals with outbreak related laboratory confirmed giardiasis [3]. Five years after the outbreak, patients who reported chronic fatigue in this questionnaire were invited to participate in a thorough clinical evaluation and screening. Fifty three individuals agreed to participate. Five patients were excluded from this study after evaluation. Two control groups were recruited; 22 individuals with normal score (=11) in the questionnaire three years after, and 10 healthy individuals not affected by the outbreak (unexposed controls) and without particular abdominal symptoms or fatigue.

In 2007, three years after a *Giardia* outbreak in Bergen, Norway, 1252 persons with stool-microscopy confirmed *Giardia* infection during the outbreak received a questionnaire regarding fatigue and abdominal complaints [3]. Five years after the outbreak, in 2009, 253 persons reporting chronic fatigue in the previous study received a mailed invitation, and 53 of these chose to participate in the present study, see Figure 1. These patients went through a clinical evaluation and were screened with a battery of routine blood tests. A total sample size of 50 was required for comparing two groups to obtain statistically significant results with a power of 80% with standardized effect size of 1 and alpha of 0.01. Due to the stratification of the patient population through clinical evaluation and questionnaires, we included the 53 patients willing to participate. The control group of 30 sex and age matched individuals included 22 *Giardia* exposed, non-fatigued questionnaire respondents and 10 healthy *Giardia* unexposed individuals. All were HIV negative and were not taking immunomodulatory or antibiotic medications. Written consent was obtained from the participants. The study was approved by the Regional Committee for Ethics in Medical Research and the Norwegian Social Society Data Services in Bergen, Norway.

### Sampling

Patient and control blood samples for exclusion of other diseases and for the purpose of this study were drawn once between 08am and 09am after overnight fast and analyzed in parallel during the same period. Fecal samples were screened by microscopy and 18S PCR [32] of feces to rule out chronic giardiasis.

### Questionnaires

On the day the blood samples were drawn, both patients and controls completed a Rome II questionnaire [5] enabling the classification of abdominal symptoms into IBS and FD or other FGID. An abdominal symptom scoring form was used to quantify abdominal symptoms [33]. Questions about abdominal complaints before the *Giardia* infection enabled identification of post-infectious FGIDs. To evaluate severity of fatigue, patients and controls also completed the Fatigue Questionnaire [30], a validated set of 11 questions addressing different aspects of fatigue.

### FGID and fatigue categorization for analysis

Patients and controls were classified according to the Rome II questionnaire with regard to FGID. Patients were clinically evaluated once, but in separate consultations with specialists in internal medicine, psychiatry and neurology. They were classified as PI-CFS or PI-idiopathic chronic fatigue (ICF) according to the 1994 Fukuda criteria [34], or as “fatigue other causes” when most plausibly due to other co-morbidities, or as “fatigue recovered” for individuals who had fully recovered from the fatigue (Morch et al., submitted).

Three different categorizations of patients and controls were formed for the analyses of the relative importance of CFS and FGID co-morbidity on immunological variables; a fatigue categorization, a FGID categorization and a combined categorization (Table 1). In the fatigue categorization, exploratory analyses of lymphocyte subsets in the small group of patients with PI-ICF (n=5) showed similarities with the PI-CFS group. The two groups were therefore analyzed together unless otherwise clearly stated.

**Table 1:** **Sex ratio and mean age in the different clinical groups after allocation of patients and controls according to Rome II FGID groups**[5]**and Fukuda CDC criteria**[34]**for the three analysis categorizations used in the present study**

				**Breakdown into recruited groups**		
	**Age (mean (SD))**	**Females (%)**	**Total participants, n**	**Exposed patients, fatigue at 3 years, n**	**Exposed controls, no fatigue at 3 years, n**	**Unexposed, healthy controls, n**
*Fatigue categorization*						
no-fatigue	40.9 (11.7)	72	32	0	22	10
CFS	45.5 (9.1)	79	19	19	0	0
ICF	37.8 (8.9)	80	5	5	0	0
Fatigue other cause	45.5 (15.2)	100	12	12	0	0
Fatigue recovered	36.0 (9.6)	58	12	12	0	0
*FGID categorization*						
no-FGID	41.8 (13.2)	78	26	5	11	10
All FGID	41.4 (10.8)	76	54	43	11	0
PI-FGID	40.8 (10.2)	76	45	36	9	0
IBS	41.7 (10.4)	80	30	27	3	0
PI-IBS	40.4 (10.4)	79	24	22	2	0
Other FGID	41.1 (11.5)	71	24	16	8	0
*Combined categorization*						
no-FGID/no-fatigue	39.9 (12.5)	74	23	2	11	10
FGID&CFS/ICF	44.3 (9.4)	78	23	23	0	0
CFS, no-FGID	35.0	100	1	1	0	0
FGID, no-fatigue	39.1 (10.1)	62	21	10	11	0
Fatigue other cause w/wo FGID	45.5 (11.5)	100	12	12	0	0

Abbreviations: *CFS*: chronic fatigue syndrome, *FGID*: functional gastrointestinal disorder, *ICF*: idiopathic chronic fatigue, *IBS*: irritable bowel syndrome, *PI*: post-infectious.

Subgroups of interest within each group are indicated by indents.

Regarding the FGID categorization, a number of patients’ symptoms did not match either the FD or the IBS criteria. These would qualify for the less well-defined FGID like functional bloating, functional diarrhea, functional abdominal pain and unspecified functional bowel disorder. For simplicity they were termed “other FGID”, and in some analyses they were grouped together with the IBS and FD groups forming an “all FGIDs” group. Three individuals had both FD and IBS and were grouped as IBS in the analysis. Some exposed non-fatigued controls also had FGID and were put together with the FGID group in some analyses. Seven of the participants had FGID-like abdominal symptoms also before their *Giardia* infection. In some analyses these were taken out in order to analyze PI-IBS and PI-FGID specifically.

In the analyses we also set up a combined categorization with one group of participants who had both FGID and CFS/ICF, one group who had FGID only, and one group with fatigue other cause with or without FGID. These groups were compared to each other and to healthy controls with neither FGID nor fatigue.

### Lymphocyte quantification

Lymphocyte subpopulation quantification was performed using the BD Multitest 6-color TBNK kit with BD Trucount Tubes for relative and absolute concentration determination (BD Biosciences, San Jose, CA, USA). The samples were prepared according to the manufacturer’s instructions and analyzed on a BD Canto II flow cytometer (BD Biosciences).

### PBMC acquisition and immunophenotyping

Peripheral blood mononuclear cells (PBMC) were isolated by density gradient separation from BD Vacutainer Na-citrate CPT tubes (BD, Franklin Lakes, NJ, USA). After harvesting, the PBMC were washed twice in PBS and cell suspensions (50μl) were stained 30 minutes in the dark using combinations of the following optimally titrated fluorescent dye-conjugated antibodies anti-CD8a-FITC, anti-CD27-FITC, anti-CD26-PE, anti-β7-PE, anti-CD45RO-PE, anti-CD4-PerCP-Cy5.5, anti-CD56-PerCP-Cy5.5, anti-CD19-PE/Cy-7, anti-CD45RA-PerCP-Cy5.5, anti-HLADR-PE/Cy7, anti-CD25-PE/Cy7 (BioLegend, SanDiego, CA, USA), anti-CD3-ECD (Beckman Coulter, Brea, CA, USA) and anti-CD127-PerCP-Cy5.5 (eBioScience, SanDiego, CA, USA). Appropriate isotype controls from the same manufacturer were used at equal concentrations. After staining, cells were washed once, resuspended in PBS-paraformaldehyde solution (1%) and analyzed the same day using a Beckman Coulter Cytomics FC 500 MPL flow cytometer. In a typical acquisition 7 × 10^4^ lymphocytes (min 2.3 × 10^4^, max 1.7 × 10^5^) were collected. The collected data were analyzed with FlowJo 7.6 software (Tree Star Inc, Ashland, OR, USA).

### Statistical analysis

Unless otherwise stated, the data are presented as mean (standard deviation (SD)). Chi-squared tests were used for categorical comparisons between groups. Lymphocyte subset data and stimulation indices were analyzed using the Kruskal-Wallis test for all groups within each categorization and then Mann Whitney U test to compare two groups. The General Linear Model was used for multivariate analyses of possible interactions and confounding factors like sex and age on lymphocyte subset levels and for correlations between these and symptom scores. Due to multiple comparisons in lymphocyte subset analyses and a high number of variables, we chose a nominal significance level of 0.01. PASW 18 (SPSS Inc, Chicago, Ill, USA) was used for statistical analysis.

## Results

Data from 48 patients (mean age 42.3 years (11.5) range19-68, females 79%), 22 exposed controls (mean age 39.8 (10.5) range 26–66, females 73%) and 10 unexposed controls (mean age 43.3 (14.3) range 22–63, females 76%) were analyzed in this study. All participated in lymphocyte quantification, but for logistical reasons not all patients and controls had all immunological tests done, numbers given in Figure 1. The numbers of patients and controls allocated into the fatigue, FGID and combined categorizations are shown in Table 1. There were no significant differences in age and male/female distributions between the groups in each categorization.

### Lymphocyte quantification

Peripheral blood lymphocyte subset results for the three analysis categorizations are shown in Table 2. We found the CD4:CD8 ratio to be reduced and the CD8 T-cell percentage to be increased in the FGID groups compared to the no-FGID control group. The same pattern was seen in the combined categorization, with elevated CD8 T-cell percentages and concentrations in both the group with FGID&CFS/ICF and the FGID, no-fatigue group.

**Table 2:** Peripheral blood lymphocyte quantification

	**CD4:CD8 ratio**	**CD3CD4 (%)**	**CD3CD4 10**^**6**^**cells/L**	**CD3CD8 (%)**	**CD3CD8 10**^**6**^**cells/L**	**CD16CD56 (%)**	**CD16CD56 10**^**6**^**cells/L**
*no-fatigue controls (n = 32)*	*2.43 (0.97)*	*51.1 (8.2)*	*990 (376)*	*22.9 (5.5)*	*440 (198)*	*11.7 (6.7)*	*210 (99)*
PI-CFS/ICF (n=24)	2.05 (0.76)	50.5 (7.0)	939 (366)	27.0 (7.0)	497 (207)	7.5 (3.5)**	130 (54)**
PI-CFS (n = 19)	2.01 (0.77)	50.3 (6.9)	912 (296)	27.5 (7.0)*	497 (200)	8.0 (3.6)*	138 (55)**
Fatigue other cause (n=12)	2.37 (1.14)	51.9 (8.1)	1132 (543)	26.1 (10.4)	635 (531)	8.7 (4.6)	186 (139)
Fatigue recovered (n=12)	1.80 (0.82)*	46.1 (10.3)	834 (318)	28.8 (9.1)*	545 (225)	10.6 (9.0)	203 (188)
*no-FGID (n=26)*	*2.74 (1.06)*	*53.3 (8.6)*	*1008 (363)*	*21.1 (5.4)*	*394 (167)*	*11.3 (7.1)*	*192 (80)*
All FGID (n=54)	1.96 (0.75)**	48.9 (7.7)*	958 (415)	27.6 (7.6)***	555 (311)**	9.1 (5.6)	176 (129)
PI-FGID (n=45)	2.00 (0.78)**	49.2 (7.9)*	949 (418)	27.1 (7.35)**	537 (313)*	8.8 (5.4)	169 (122)
PI-IBS (n=23)	1.97 (0.91)**	49.5 (8.8)	988 (463)	28.2 (7.1)**	592 (376)*	8.1 (5.0)*	157 (111)
*no-FGID/no-fatigue controls (n=23)*	*2.65 (1.05)*	*52.4 (8.8)*	*974 (380)*	*21.5 (5.5)*	*389 (171)*	*11.8 (7.6)*	*194 (86)*
FGID&CFS/ICF(n=23)	2.01 (0.75)*	50.2 (7.0)	923 (366)	27.3 (6.9)**	500 (211)	7.4 (3.5)**	127 (53)*
FGID, no fatigue (n=21)	1.82 (0.65)**	46.9 (8.5)*	924 (356)	27.9 (7.3)**	548 (214)**	11.0 (7.0)	223 (156)
Fatigue other cause w/wo FGID (n=12)	2.37 (1.14)	51.9 (8.1)	1133 (543)	26.1 (10.4)	635 (531)	8.7 (4.6)	186 (140)

* p < 0.05 level compared to the control group (in italics) in each categorization.

** Significant at p<0.01 level compared to the control group (in italics) in its categorization.

*** Significant at p<0.001 level compared to the control group (in italics) in its categorization.

CD4:CD8 T-cells ratio, CD4 and CD8 T-cell subsets, CD16CD56 natural killer cells percentages and concentrations by analysis categorizations and relevant subgroups. All values are given as mean (SD). Data for the subgroups of PI-CFS, PI-FGID and PI-IBS are also given (indented).

As has been reported by others [35,36] the NK-cell percentage measurements were significantly affected by sex, with men having higher levels than women (p=0.006). NK-cell levels also increased with increasing age (p=0.033). Correcting for this using multivariate analysis showed that PI-CFS/ICF patients had a lower percentage of NK-cells than the no-fatigue controls (p=0.005) when analyzing within the fatigue categorization. Also in the combined categorization the FGID&CFS/ICF patient group had lower percentage (p=0.006) and concentration (p=0.045) of NK-cells. The percentage and concentrations of CD3 and CD4 T-cells and CD19 B-cells were similar in all groups tested.

**Figure 2:**
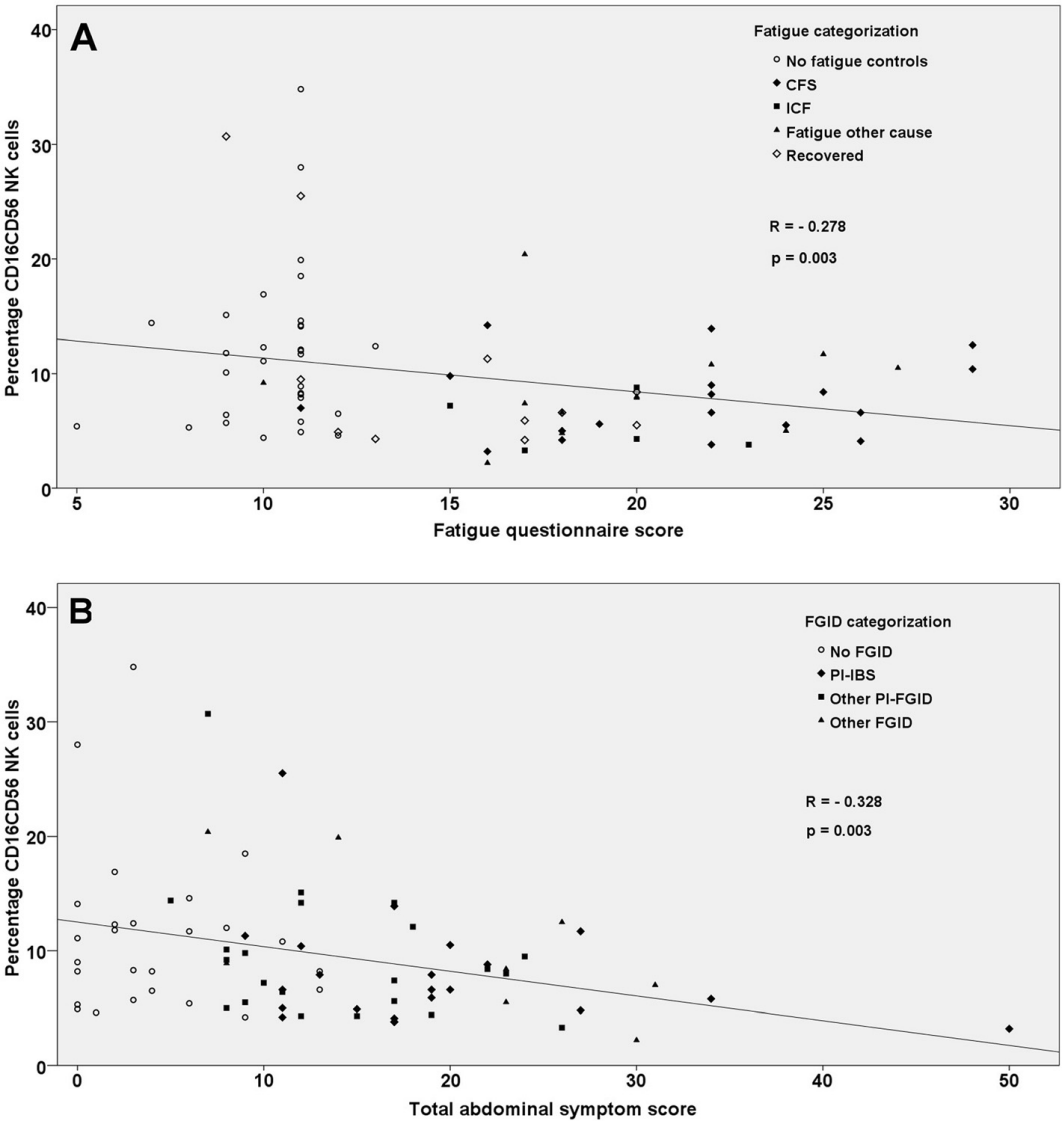
**NK-cell levels and symptoms correlations.** Correlation plots between the percentage of peripheral blood CD16CD56 NK-cells and fatigue symptom score (**A**) as recorded by the fatigue questionnaire [30] and total abdominal symptoms score (**B**) (including the six symptoms nausea, early satiety, bloating, abdominal pain, constipation and diarrhoea, where patients graded their symptoms on an ordinal scale from 0 to 10 with 0 = no symptoms and 10 = severe symptoms).

A significant correlation was found between the fatigue scores and abdominal symptom scores (R=0.421, p<0.001). NK-cell percentage correlated significantly with both fatigue scores (p=0.003) and with abdominal symptom scores (p=0.003) (Figure 2) and so did NK-cell concentrations with p=0.01 and p=0.009, respectively. Levels of CD8 T-cells did not show any correlation with symptoms.

### Immunophenotyping

CD4 and CD8 T-cells were analyzed for expression of activation markers HLA-DR, CD25, CD26, and CD27 and CD45RO. CD56^+^ NK-cells were analyzed for CD26. CD19^+^ B-cells were analyzed for activation markers CD25, CD26 and CD27. Additionally, we analyzed percentage of gut-homing integrin β7 positive T-cells, B-cells and CD4^+^CD25^++^CD127^neg^ T-regulatory cells.

The percentage of CD26 positive CD3 T-cells in the “all FGID” group (78.5%(9.4)) was insignificantly decreased compared to the control group without FGID (83.4%(6.4)) in multivariate analysis (p=0.036). We did not find any significant differences along the FGID or CFS categorizations regarding the percentage of CD56^dim^ or CD56^bright^ NK-cells or for CD26 positive subpopulations of these NK-cell subsets. Nor did we find any significant differences between groups for activation markers and memory markers in T-cells, B-cells or in the percentage of gut-homing integrin β7 positive T-cells and B-cells or CD4^+^CD25^++^CD127^-^ Treg cells (data not shown).

## Discussion

In this study we classified patients and controls with FGID according to Rome II criteria and by the Fukuda CDC criteria for CFS and ICF regarding fatigue. A wide range of peripheral blood lymphocyte subsets reported to be altered in CFS and FGID were analyzed.

### CD8 T-cells and CD4:CD8 ratio

Searching the literature regarding CD4 and CD8 T-cell subsets in FGID we found two studies which did not find differences in these T-cell subsets [15,16] and two studies with lowered CD4/CD8 ratio in IBS patients; one due to high total CD8 cells [37] and one due to low CD4 cells [38]. While CD4 and CD8 T-cell numbers and percentages may fluctuate considerably within an individual over time, the CD4:CD8 ratio is found to be relatively stable [39]. We found the CD4:CD8 ratio to be low due to increased CD8 T-cell numbers in our PI-FGID study population. Similar peripheral blood T-cell patterns are reported in patients suffering from a number of viral diseases like mononucleosis, dengue, RSV and cytomegalovirus infection and herpes simplex recrudescence as well as in chronic toxoplasmosis infection [39,40]. A low grade ongoing immune response against reactivated viruses is a hypothesis of CFS morbidity [41].

Without controlling for FGID co-morbidity, and including the small group of patients recovered from fatigue, the elevated CD8 levels might be interpreted as supportive of this hypothesis. Due to the categorization of the co-morbidity, the elevated CD8 level was seen to be a feature of PI-FGID, rather than PI-CFS. Whether the finding is a cause or effect of the *Giardia* induced FGID needs further exploration.

CD8-T-cells have been shown to be responsible for the mucosal injury and temporary loss of disaccharidase activity in acute giardiasis [28]. Our finding of elevated CD8 T-cell levels in post-giardiasis FGID patients may indicate an association between the mucosal injury during the acute infection and the observed prolonged syndromes.

### NK-cells in FGID and CFS

NK-cells do not seem to play a role in clearing acute *Giardia* infection [29]. No previous study has evaluated their possible role in post-giardiasis IBS or CFS. Two studies in general IBS populations found peripheral blood CD56^+^ NK-cells levels to be similar to healthy controls [16,42]. A post-prandial decrease in NK-cells in IBS patients relative to controls has also been described [43].

The majority of studies of NK-cells in heterogeneous CFS populations have not found differences in NK-cell levels [11,12]. However, two studies have found significantly reduced NK-cell levels in both PI-CFS and non-PI-CFS patients [19,44]. One small study has shown NK-cell levels to return to normal after successful recovery in the PI-CFS group [45].

In our study, fatigue scores correlated with NK-cell levels, and the patients who had recovered from chronic fatigue showed NK-cell levels comparable to the non-fatigue control group. These data suggest that decreased NK-cell levels are associated with ongoing fatigue in post-giardiasis PI-CFS. As we also found NK-cell levels to be correlated with abdominal symptom scores, an alternative explanation might be that NK-cell levels play a role in sensitization or interpretation of sensory stimuli.

### Activation markers and subsets

We could not replicate the elevated levels of CD26 expression in T and NK-cells as a robust marker of CFS reported by Fletcher et al. [13]. Instead, there was a tendency towards lower CD26^+^ T-cells in FGID patients.

A study in CFS patients recently reported higher levels of CD4^+^CD25^++^FoxP3^+^ cells [12]. We found no difference in regulatory T-cells with regard to fatigue categorization as measured by CD4^+^CD25^++^CD127^-^ cells, and also not with regard to FGID categorization. Also a previous study regarding regulatory T-cells in FGID patients did not find any such difference [14].

### Cautionary remarks

NK-cell levels are influenced by sleep and depression [46]. We did not control for poor sleep, but no patients in the CFS/ICF group had clinical depression. NK-cells are also known to fluctuate considerably with exercise, but the resting immune function evaluated in this study, is not very different in athletes compared to non-athletes [47]. The cytotoxic activity of NK-cell has been more consistently found decreased in CFS studies, but may be a bystander effect as it has been shown to be decreased in chronic stress like unemployment, while NK-cell concentration was not [48].

Out of the 253 invited persons, 53 consented to participate. We believe the main reason for the low participation rate was that the basis for invitation to participate was fatigue score at two years previously and that many had recovered during this time span. Another reason may be that the study logistics with five appointments for clinical evaluations and testing was too demanding for some. However, a chance for bias regarding increased health seeking behavior in the participants cannot be excluded. Also, we cannot fully exclude the contribution of an additional viral trigger in the development or perpetuation of fatigue symptoms.

## Conclusions

In the patients who developed PI-FGID and PI-CFS after giardiasis, we found significantly increased CD8 T-cell levels in patients with FGID and reduced levels of NK-cells in CFS patients. There was a positive correlation between fatigue scores and abdominal symptom scores in the study population, and the severity of these symptom scores correlated negatively with NK-cell levels. The findings suggest an immunological abnormality in these patients and the potential and relative importance of NK-cells and CD8 T-cells in the co-morbid conditions FGID and CFS should be further explored.

## Abbreviations

CFS: Chronic fatigue syndrome; FD: Functional dyspepsia; FGID: Functional gastrointestinal disorder; GLM: General linear model; ICF: Idiopathic chronic fatigue; IBS: Irritable bowel syndrome; NK: Natural killer; PI: Post-infectious.

## Competing interests

The authors declare that they have no competing interests. This work was supported by The Western Norway Regional Health Authority and the University of Bergen. Data were analyzed and evaluated independently by the authors, without any interference from the funding institution.

## Authors’ contributions

Clinical evaluation of patients was done by KM, HN, ACR, and JEB. Additional data collection and analyses were performed by KH and KM. Laboratory work and flow-cytometry analyses were done by KH, EK, and SS. NL supervised all parts of the study. TH assisted in planning and all authors assisted in preparation of the manuscript. All authors read and approved the final manuscript.

## Pre-publication history

The pre-publication history for this paper can be accessed here:

http://www.biomedcentral.com/1471-2334/12/258/prepub
